# Supplementary material for: Carotid Plaque Vulnerability Diagnosis by CTA versus MRA: A Systematic Review
Source: Diagnostics (Basel). 2023 Feb 9;13(4):646. doi: 10.3390/diagnostics13040646 (PMC9955971; doi:10.3390/diagnostics13040646)
Supplement: Supplementary file 1 [file diagnostics-13-00646-s001.zip › Supplementary Table S1 - PICO.pdf]

Supplementary Table S1. PICO Model

P	Patient, population or problem	Patients with symptomatic (stroke, TIA, amaurosis fugax) or asymptomatic carotid artery stenosis
I	Intervention, prognostic factor or exposure	Carotid arteries' characteristics detected in CTA
C	Comparison of intervention	Carotid arteries' characteristics detected in MRA
O	Outcome you would like to measure or achieve	Carotid plaque vulnerability, including IPH, LRNC, TFC, plaque neovascularization, inflammation, ulceration, calcification, or thrombus
	What type of question are you asking?	Does CTA presented similar accuracy with MRA (reference study) for the detection of carotid plaque vulnerable characteristics in symptomatic and asymptomatic patients?
	Type of study you want to find	Observational studies (Randomized, Non-randomized)

Footnote: P.I.C.O. (patient; intervention; comparison; outcome) model was used to define the clinical questions and clinically relevant evidence in the literature, TIA; Transient Ischemic Attack, CTA; Computed tomography angiography, MRA; Magnetic resonance angiography, IPH; Intra-plaque hemorrhage, LRNC; Lipid-rich necrotic core, TFC; Thin fibrous cap
