# Supplementary material for: Carotid Plaque Vulnerability Diagnosis by CTA versus MRA: A Systematic Review
Source: Diagnostics (Basel). 2023 Feb 9;13(4):646. doi: 10.3390/diagnostics13040646 (PMC9955971; doi:10.3390/diagnostics13040646)
Supplement: Supplementary file 1 [file diagnostics-13-00646-s001.zip › Supplementary Table S2 - Search Strategy.pdf]

Supplementary Table S2. Search Strategy
Comparative studies on humans, English Studies, RCT and Observational studies
Subject Area: Medicine
Time Period: Inception-2022
Exclusion criteria: 1. Case reports 2. Case Series 3. Studies reporting solely on one or the other method of imaging 4. Non-comparable studies 5. Reviews

PUBMED (HITS: 2512)	((((((stroke) OR (stroke[MeSH Terms])) OR ((TIA) OR (TIA[MeSH Terms]))) OR ((transient ischemic attack) OR (transient ischemic attack[MeSH Terms]))) OR ((amaurosis fugax) OR (amaurosis fugax[MeSH Terms]))) AND (((computed tomography angiography) OR (computed tomography angiography[MeSH Terms])) OR ((magnetic resonance angiography) OR (magnetic resonance angiography[MeSH Terms])))) AND ((((((((((((intraplaque hemorrhage) OR (intraplaque hemorrhage[MeSH Terms])) OR (IPH)) OR (IPH[MeSH Terms])) OR ((((lipid-rich necrotic core) OR (lipid-rich necrotic core[MeSH Terms])) OR (LRNC)) OR (LRNC[MeSH Terms]))) OR ((((Neovascularization) OR (Neovascularization[MeSH Terms])) OR (Neovascularisation)) OR (Neovascularisation[MeSH Terms]))) OR ((Inflammation) OR (Inflammation[MeSH Terms]))) OR ((Ulceration) OR (Ulceration[MeSH Terms]))) OR ((calcification) OR (calcification[MeSH Terms]))) OR ((thrombus) OR (thrombus[MeSH Terms]))) OR ((((thin-fibrous cap) OR (thin-fibrous cap[MeSH Terms])) OR (TFC)) OR (TFC[MeSH Terms]))) OR ((carotid plaque vulnerability) OR (carotid plaque vulnerability[MeSH Terms])))
SCOPUS (HITS: 663)	"stroke" OR "transient ischemic attack" OR "amaurosis fugax") AND ("computed tomography angiography" OR "magnetic resonance angiography") AND ("intraplaque hemorrhage" OR "lipid-rich necrotid core" OR "carotid plaque neovascularization" OR "carotid plaque

	neovascularisation" OR "carotid plaque inflammation" OR "carotid plaque ulceration" OR "carotid plaque calcification" OR "carotid plaque thrombus" OR "thin fibrous cap" OR "carotid plaque vulnerability"
CENTRAL(HITS: 127)	#1 stroke<br>#2 [mh "stroke"]<br>#3 transient ischemic attack<br>#4 [mh "transient ischemic attack"]<br>#5 amaurosis fugax<br>#6 [mh "amaurosis fugax"]<br>#7 TIA<br>#8 [mh "TIA"]<br>#9 computed tomography angiography<br>#10 [mh "computed tomography angiography"]<br>#11 magnetic resonance angiography<br>#12 [mh "magnetic resonance angiography"]<br>#13 intraplaque hemorrhage<br>#14 [mh "intraplaque hemorrhage"]<br>#15 lipid rich necrotic core<br>#16 [mh "lipid rich necrotic core"]<br>#17 neovascularization<br>#18 [mh "neovascularization"]<br>#19 neovascularization<br>#20 [mh "neovascularisation"]<br>#21 inflammation<br>#22 [mh "inflammation"]<br>#23 calcification<br>#24 [mh "calcification"]<br>#25 ulceration<br>#26 [mh "ulceration"]<br>#27 thrombus<br>#28 [mh "thrombus"]<br>#29 vulnerable carotid plaque<br>#30 [mh "vulnerable carotid plaque"]<br>#31 {OR #1-#8}<br>#32 {OR #9-#12}<br>#33 {OR #13-#30}<br>#34 {AND #31-#33}

Total Hits: 3302
Duplicate Screening: 3128
Automatic Tool Duplicate Removal: 135
By Hand Duplicate Removal: 39
Title-Abstract Screening: 51

Data Overlap Screening: 51
Full-Text Screening: 5
